# Confined Vacuum Resonances as Artificial Atoms with Tunable Lifetime

**DOI:** 10.1021/acsnano.2c04574

**Published:** 2022-07-11

**Authors:** Rasa Rejali, Laëtitia Farinacci, David Coffey, Rik Broekhoven, Jeremie Gobeil, Yaroslav M. Blanter, Sander Otte

**Affiliations:** Department of Quantum Nanoscience, Kavli Institute of Nanoscience, Delft University of Technology, Lorentzweg 1, Delft 2628 CJ, The Netherlands

**Keywords:** scanning tunneling microscopy (STM), scanning tunneling spectroscopy (STS), electronic lattices, field-emission resonances, negative differential resistance, electronic lifetime of confined states, resonant transport

## Abstract

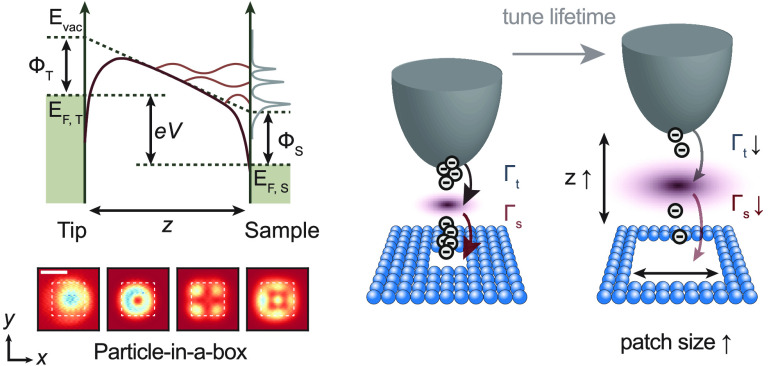

Atomically engineered artificial lattices are a useful tool for simulating complex quantum phenomena, but have so far been limited to the study of Hamiltonians where electron–electron interactions do not play a role. However, it is precisely the regime in which these interactions do matter where computational times lend simulations a critical advantage over numerical methods. Here, we propose a platform for constructing artificial matter that relies on the confinement of field-emission resonances, a class of vacuum-localized discretized electronic states. We use atom manipulation of surface vacancies in a chlorine-terminated Cu(100) surface to reveal square patches of the underlying metal, thereby creating atomically precise potential wells that host particle-in-a-box modes. By adjusting the dimensions of the confining potential, we can access states with different quantum numbers, making these patches attractive candidates as quantum dots or artificial atoms. We demonstrate that the lifetime of electrons in these engineered states can be extended and tuned through modification of the confining potential, either via atomic assembly or by changing the tip–sample distance. We also demonstrate control over a finite range of state filling, a parameter which plays a key role in the evolution of quantum many-body states. We model the transport through the localized state to disentangle and quantify the lifetime-limiting processes, illustrating the critical dependence of the electron lifetime on the properties of the underlying bulk band structure. The interplay with the bulk bands gives rise to negative differential resistance, leading to possible applications in engineering custom atomic-scale resonant tunnelling diodes, which exhibit similar current–voltage characteristics.

Artificial lattices serve as quantum simulators for realizing and studying fundamental properties of real materials, with the advantage that the relevant interactions can be precisely controlled. While different experimental approaches, such as using ultracold atoms,^1^ optical lattices,^2,3^ or trapped ions,^4^ have been successfully implemented in the study of artificially constructed systems, atom manipulation casts scanning tunneling microscopy (STM) as a particularly appealing platform: The scanning probe framework allows for creating and characterizing the electronic properties of 2D artificial matter on the atomic scale.^5^ Typically, atomic impurities are patterned to construct a potential landscape that mimics a specific physical system, with the aim of studying model Hamiltonians. This approach has led to the realization of a wide range of quantum states in, for instance, Dirac materials like the Lieb lattice^6,7^ and artificial graphene,^8,9^ as well as higher order topological insulators,^10,11^ among others.^12−16^ These studies offer rare insight into the parameters that govern the electronic behavior of these systems, but they are restricted by the short electron lifetime of the constituent artificial atoms to the limiting case in which electron–electron interactions do not play a role. Additionally, short electron lifetimes limit the available energy resolution; the most popular STM approach so far, which relies on confining surface states, lacks flexibility in tuning this parameter.^17−19^

Here, we explore a platform for realizing artificial lattices, based on confining field-emission resonances (FERs): a class of quantized electronic states localized in the vacuum, between the surface and the probe tip, that arise in the high bias regime, i.e., exceeding the sample work function. We show that confining potentials can be engineered to enable the study of states with different orbital character,^9,20,21^ with precise control over the energy and quantum numbers of the states. We study the electron lifetime of these states, and demonstrate that we can finely tune it, and consequently, to some extent, the average occupation, by adjusting the tip height or patch dimensions. The ability to tune the lifetime and occupation of artificial atoms is a prerequisite for simulating many-body quantum states driven by electron–electron interactions. We also observe specific voltage–current characteristics, namely, negative differential resistance, which are analogous to those of resonant tunneling diodes,^22^ making the confined FERs also suitable to possible applications in creating customizable, atomic-scale diodes.

## Results and Discussion

**Figure 1:**
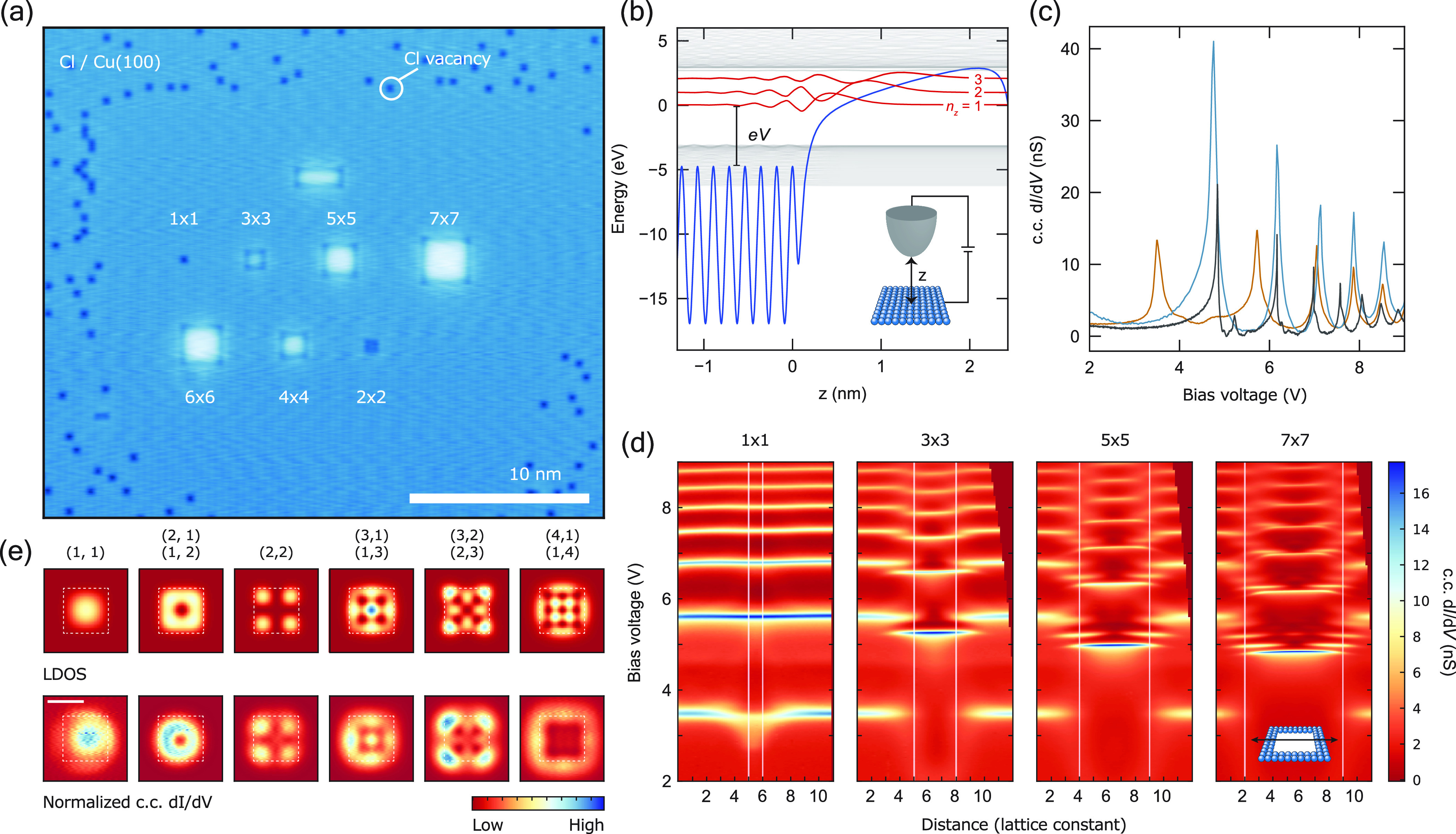
Confinement of field-emission resonances. (a) STM constant-current topography (600 mV, 300 pA) of square, atomically assembled patches of Cl vacancies, with sizes indicated in unit cells. (b) Potential landscape (blue) between sample (left) and tip (right) for a finite bias voltage *V*. Among the wave functions (gray) calculated for this potential are the first three field-emission resonances (red). Inset: schematic of the tip–sample junction. (c) Constant-current differential conductance spectra acquired for bare Cu(100) (blue, 250 pA), the chlorine monolayer (orange, 100 pA), and the center of the 7 × 7 patch (black, 100 pA). The first peak on the chlorine monolayer (3.5 V), being below the surface work function, corresponds to an image-potential state. (d) Stacked constant-current (100 pA) differential conductance spectra taken along a line crossing the center of each patch (shown in inset), with the corresponding patch size indicated (top). A correction is applied to the data to rectify the asymmetry of the tip electric field (see Figure S5). White lines indicate the patch boundaries. (e) Calculated LDOS of the particle-in-a-box states (|Ψ|^2^), obtained using a finite well model (top row). Normalized^38^ constant-current (100 pA) differential conductance maps acquired for the 7 × 7 patch at the resonance energies of the first principal FER (*n*_*z*_ = 1, (*n*_*x*_, *n*_*y*_) = (1,1)) and the following subresonances. White squares delineate the spatial extent of the simulated potential well (top row) and the physical patch (bottom row).

We use atom manipulation of single vacancies in the chlorine-terminated Cu(100) surface to engineer lateral confinement of field emission resonances. By coordinating chlorine vacancies, which are easily manipulable and thus suited to large scale atomic assembly,^6,13−15,23^ adjacent to each other, we construct patches of exposed copper, surrounded by areas of homogeneous, monolayer chlorine coverage (Figure 1a). As shown in Figure 1c, the bare and chlorinated Cu(100) surfaces host FERs at bias voltages exceeding the local work function, at 4.6 V^24^ and 5.7 V,^25^ respectively. Although these spectra are obtained at slightly different current set points, we can expect this discrepancy to have a minor effect in the observed spectroscopic features.^26^

These resonances can be readily modeled with a one-dimensional potential in the out-of-plane direction (Figures 1 and S4). The work function difference between the two surfaces results in a shift in the measured resonance energies (Figure 1c), in accordance with previous studies.^27−30^

Spectroscopy acquired at the center of the 7 × 7 patch (dimensions defined in unit cells of the chlorine lattice) exhibits additional resonances, in comparison to the bare and chlorinated Cu(100) surfaces (Figure 1c). As shown in Figure 1d, these additional resonances belong to a series of subresonances following each primary FER and can in fact be resolved for each primary FER, up to and including the fourth primary resonance. We use the principal quantum number *n*_*z*_ to describe the primary FERs. The additional modes are only observed above the energy of the first resonance on bare Cu(100) (Figure 1c). The full in-plane structure of the confined modes for the larger patches is best visualized by differential conductance maps taken at voltages corresponding to the subresonances of the first FER on the 7 × 7 patch, as shown in Figure 1e. The observed states can be recognized as two-dimensional particle-in-a-box modes, with quantum numbers *n*_*x*_ and *n*_*y*_, and can be accurately reproduced by the eigenstates of a finite potential well (see Figure S3). Similar to previous works,^21^ the nodal patterns of the first three modes are analogous to the orbitals of a two-dimensional atom, with the first state corresponding to the s-like state and the second to the p-like and subsequently the d-like state. Finally, we note that the energy of the FERs depends on the patch size: As the patch size is increased, the FER energy shifts down, tending toward the limit of bare Cu(100). All in all, the assembled patches can be seen as atomically precise potential wells, wherein the energy, spacing, and order of the states can be tuned by adjusting the shape and size of the confining potential. We note that the single vacancy^6,13−15^ stands out as an exception, as the finite screening length prohibits the necessary change in the local work function on such small length-scales: As such, the vacancy acts as a scattering center, rather than a confinement potential.

**Figure 2:**
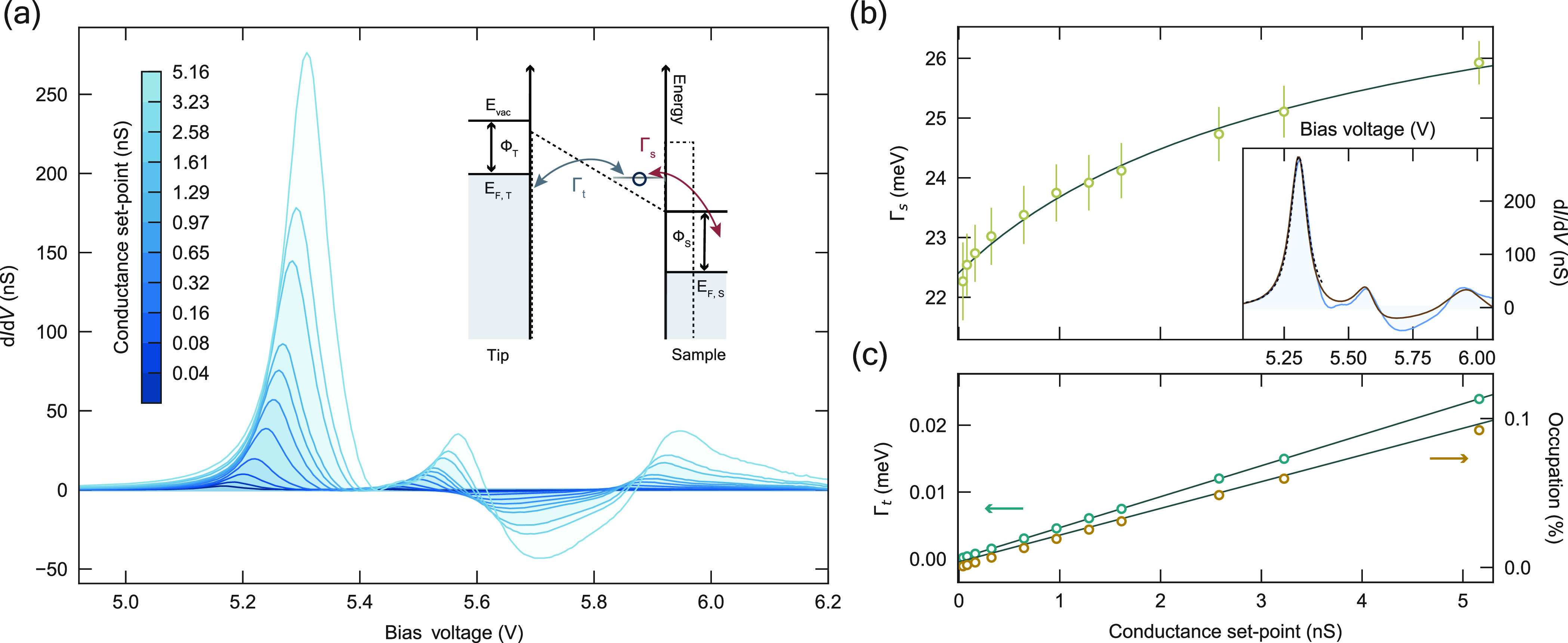
Extracting tip and sample decay rates. (a) Constant-height differential conductance spectra obtained at the center of the 5 × 5 patch for a range of conductance set points ((250 pA → 32 nA), 6.2 V, corresponding to a ∼0.3 nm change in tip–sample distance). Inset: schematic of the double-barrier potential (dotted line) implemented in the rate equations, with the indicated decay rates to the tip and sample, Γ_*t*_ and Γ_*s*_. (b) Inset: constant-height differential conductance (light blue, shaded) acquired at the center of the 5 × 5 patch (32 nA, 6.2 V). Calculated d *I*/d*V* using a resonant tunneling model for a single level (black, dotted line) or several, independent levels (brown, solid line). (b, c) Sample (b, yellow circles) and tip (c, green circles) decay rates extracted for the first principal resonance as a function of conductance set point, fit (solid gray line) to an inverse natural logarithm and a line, respectively. The tip decay rate is evaluated at the energy of the peak of the first principal field emission resonance. (c) Average occupation versus conductance set point (orange circles) and the corresponding linear fit (solid gray line).

In order to characterize the electron lifetime, we consider the transport through these confined states: Two electron baths, one on the tip side and another on the sample side, act as decoherent sources, the contributions of which we can disentangle by investigating the evolution of the differential conductance spectra as a function of conductance set point, as shown in Figure 2a. With increasing conductance set point, we observe a slight shift in the energy of the FERs, which is explained by the increased out-of-plane confinement (Figure 1b), as well as the appearance of negative differential resistance (NDR). The appearance of NDR at high conductance set points gives us qualitative insight into the coupling of the resonances with the substrate and tip.

We consider a transport model describing the resonant tunneling of independent electrons from (to) the tip and sample through a level localized between the two potential barriers (Figure 2a, inset, and Supporting Information section 1). In this framework, the current through a single resonance is given by
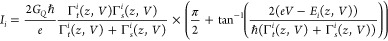
1where the quantum of conductance is *G*_*Q*_ = *e*^2^/(πℏ), Γ_*t*_^*i*^ and Γ_*s*_^*i*^ are, respectively, the tip and sample decay rates for the *i-*th resonance, and *E*_*i*_ is its energy, whose shift with bias voltage we will initially neglect for simplicity. In general, the tip and sample decay rates are both distance and voltage dependent. For the former, this dependence is derived by considering the transmission through the tunnel barrier. The sample decay rate, however, encapsulates an effective barrier that depends on the surface band structure, and the relationship between Γ*_s_* and *V* is nontrivial. We approximate this dependence as either constant or linear, depending on the width of the voltage window we consider. The differential conductance, in turn, can be obtained by differentiating the current with respect to voltage, and contains terms that scale with the derivatives of the decay rates and the energy of the resonance (see Supporting Information equation S10 for the full expression).

We can gain quantitative insight into the tip and sample decay rates by focusing strictly on the first principal FER (Figure 2b, inset): This allows us to drastically reduce the number of free variables to a single resonance, and consequently to meaningfully account for the effects of the changing level *E*_0_; additionally, we simplify Γ*_s_*(*V*) to a constant in the narrow voltage range around the resonance. By fitting the measured differential conductance at each conductance set point to our model, we can extract a value for the tip and sample decay rates as a function of conductance set point (Figure 2b, c).

In Figure 2b, we see that Γ*_s_* increases with the conductance set point, which can be related to the FER wave function: In general, decay to the bulk is governed by the overlap of the vacuum-localized state to the substrate, which is in turn determined via the penetration of the state into the bulk, the evanescent tail of the bulk states into the vacuum, and the diminished electronic screening in the area between the surface and the vacuum.^31,32^ Bringing the tip closer causes a redistribution of the weight of the wave function toward the surface, rendering the scattering channels to the bulk more efficient^33^ and leading to an increase in Γ*_s_*. More precisely, we consider that the sample decay rate should scale linearly with the wave function overlap of the FER with the sample,^31^ and for simplicity, we assume its increase to be inversely proportional to the tip–sample distance. Given the exponential dependence of current with distance, we thus expect an inverse logarithmic dependence of the sample decay rate on the conductance set point. The fit in Figure 2b shows that this simple relation appropriately describes the change in Γ_*s*_.

The evolution of the tip decay rate with conductance set point is straightforward: This rate should scale exponentially with the tip–sample distance, meaning it should be linear with the conductance set point and intercept with the origin, as we see in Figure 2c. Importantly, the changes in the decay rates impact the overall occupation of the state. The occupation is determined by the ratio of the tip decay rate to total decay rate Γ*_t_* + Γ*_s_*, meaning that the occupation of the state can be tuned via the tip-height, as shown in Figure 2c: The occupation linearly increases with the conductance set point. In effect, this means that the competing factors determining the time-average occupation, the rate of tunneling electrons versus the increase in the lifetime-limiting rate, Γ*_s_*, result in the state filling increasing as the tip is brought closer.

We now extend our scope to account for transport through the higher energy states, around 5.6 V (*n*_*z*_ = 1, (*n*_*x*_, *n*_*y*_) = (2, 1), (1, 2)) and 6 V ((3, 1), (1, 3)), respectively. To do so, we assume the resonances are independent, i.e., that the total current is determined by the sum of the currents *I*_*i*_ through each resonance; additionally, we explicitly account for the voltage-dependence of Γ*_s_*(*V*) as linear to first approximation. As seen in Figure 2b (inset), our model successfully reproduces the key features of the measured differential conductance over the entire voltage range, with, in particular, the presence of NDR between ∼5.6 and 6 V. In this window, we find dΓ*_s_*/d*V* < 0. In fact, we find it is necessary to have a decreasing sample decay rate with increasing voltage to engender NDR, indicating once again that the decay path to the sample crucially depends on the electronic wave function of the FER.

While the decay rates can be tuned by changing the out-of-plane confinement of the wave function, the in-plane confinement plays the dominant role in setting an upper bound on the lifetime. Typically, field-emission resonances are delocalized (Bloch-like) in the directions parallel to the surface and thus form bands.^26^ In that case, the electron lifetime is affected by interband scattering, wherein the excited electron escapes into the metal (sample or tip), or scatters with an electron in a different band, and intraband scattering, in which case the electron changes velocity.^31,34^ We can expect the introduction of lateral localization to affect decay through these channels in two opposing ways: The increased confinement causes the bands to split into quantized states, strongly attenuating intraband decay, while the simultaneous broadening of the *k*-space distribution increases the available interband decay paths to the bulk. We assess the degree to which the in-plane confinement precisely affects the lifetime by investigating the transport characteristics of different sized patches.

**Figure 3:**
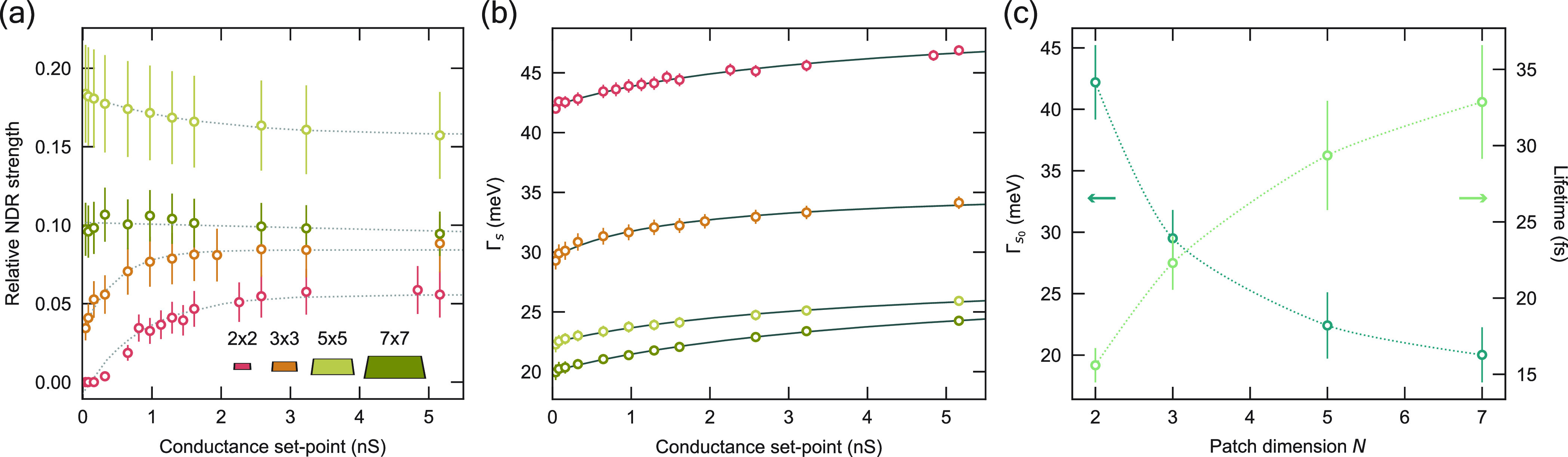
Tuning of the lifetime. (a) Relative strength of the negative differential resistance as a function of conductance set point for patches of various size. Colors correspond to illustration in the inset. Dotted lines are guides to the eye. (b) Conductance-dependence of the sample decay rate for corresponding patch sizes fit to an inverse natural logarithmic function (gray solid lines). (c) Extrapolated value of the sample decay rate for zero set point conductance (blue circles) and the corresponding lifetime (green circles) for each patch size. Dotted lines are guides to eye.

Carrying out the same conductance-dependent measurements (see Figure S6), we see a marked change in the relative strength of the NDR based on the dimensions of the confining patch, as shown in Figure 3a. The relative NDR strength, which we define as the ratio of negative area to the total area under the differential conductance spectrum, stays fairly constant as a function of conductance set point for patches of larger size, such as the 7 × 7 and 5 × 5. In contrast, the smallest patch (2 × 2) does not exhibit any NDR at low conductance set points; at a conductance set point of ∼0.5 nS, the relative NDR strength becomes nonzero and monotonically increases thereafter. The same general trend holds for the 3 × 3: exponentially increasing NDR strength with increasing conductance set point. In fact, the NDR is directly related to the change in the sample decay rate as a function of voltage, and we can see this variance in Γ*_s_* in the strength and conductance-dependent behavior of the NDR for the different patches.

As before, to quantify the change in the sample decay rate, we extract Γ_*s*_ by fitting eq 1 to the first principal FER of each patch for a discrete range of conductance set points (Figure 3b). We see that both the magnitude of the sample decay rate, and its rate of change over this conductance set point range, vary according to patch size. The electrons localized above the smallest patch experience the largest sample decay rates, meaning that scattering to the bulk becomes more efficient due to the increased spatial confinement.

The lifetime of these localized electrons, τ, is determined by the tip and sample decay rates, such that τ^–1^ = Γ_*s*_^–1^ + Γ_*t*_^–1^. The tip contribution exponentially tends to zero as a function of the tip–sample distance, meaning the intrinsic lifetime (at zero conductance set point, namely, when the tip is infinitely far away) is determined by the sample decay rate at zero conductance. Approximating the lifetime by the line width of the resonance is not valid here, as the potential in the out-of-plane direction changes as we carry out spectroscopy, leading to a changing resonance energy as a function of the applied voltage that artificially broadens the peak.

As shown in Figure 3c, the extracted lifetimes monotonically increase as a function of patch size up to *N* = 7, the maximum patch dimension studied in this work. Notably, the lifetime for the confined states is roughly 2–4 times longer than the lifetime of the first resonance on bare Cu(100), extracted using the same method and in fair agreement with previously reported values (see Figure S2). This also indicates that there must be a patch size with an optimally long lifetime, after which τ begins decreasing with patch size, tending toward the freely propagating Cu(100) limit. Indeed, the degree to which the confinement prohibits the different decay paths at play is ultimately a delicate balance: The smaller the patch, the fewer states available for scattering between different resonances but the larger the *k*-space overlap with the bulk states. Notably, the lifetime-limiting rate for all the patches shown here is Γ_*s*_, which in our case is approximately 3 orders of magnitude larger than the tip decay rate Γ_*t*_ (Figure 2c).

**Figure 4:**
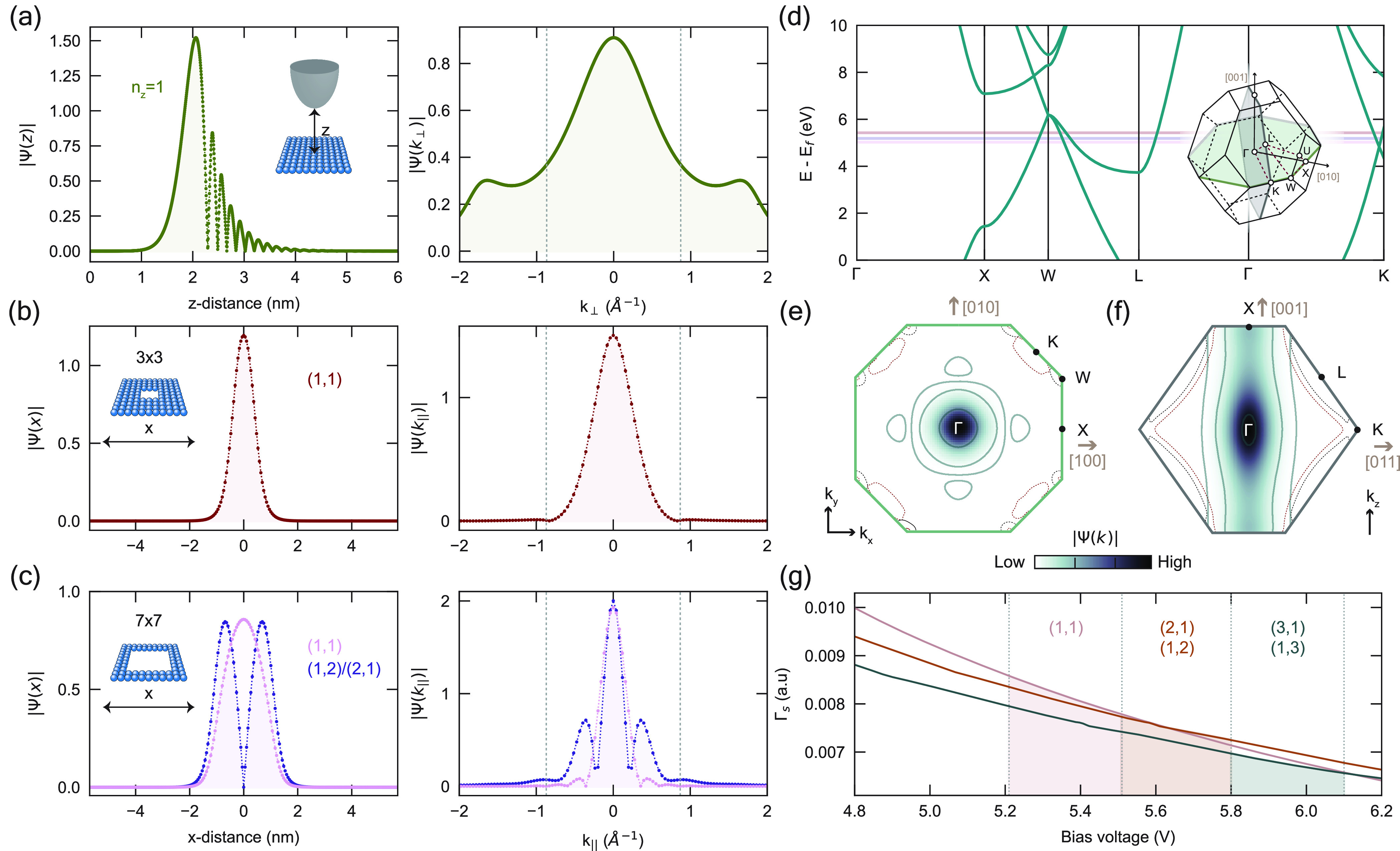
Distribution in *k*-space. (a) Calculated out-of-plane component of the real space wave function |ψ(*z*)| for the first principal FER *n*_*z*_ = 0 (left) and the corresponding Fourier transform (right) at a tip–sample distance *z* = 2.4 nm. (b, c) Calculated in-plane component of the real space wave function (left) for the (b) 3 × 3 and (c) 7 × 7 patches, showing the first (*n*_*x*_, *n*_*y*_) = (1, 1) ((b) red; (c) pink) and second (1, 2), (2, 1) ((c), purple) modes, with the corresponding Fourier transforms (right). Dotted lines indicate ± π/*a* bounds. (d) Bulk band structure of Cu along high-symmetry lines, with the experimental resonance energy of the (1, 1) state for the 3 × 3 (red) and 7 × 7 (pink), as well as the (1, 2)/(2, 1) state of the latter (purple), denoted by solid lines. Inset: schematic of the first Brillouin zone of Cu. (e, f) Intensity of the 3 × 3 wave function in *k*-space across Brillouin zone slices indicated in inset of (d). Solid contour lines delineate an order of magnitude change in the intensity. Corresponding DFT-calculated constant-energy isolines shown for bulk Cu bands, taken 5 V (black line) and 6 V (red line) above the Fermi level. (g) Calculated sample decay rate as a function of bias voltage, shown for the first three resonances probed in the center of a 5 × 5 patch, corresponding to the (1, 1) (mauve line), (2, 1)/(1, 2) (brown line), and (3, 1)/(1, 3) (gray line) modes. The shaded areas correspond to the voltage range in which the respective modes are typically measured, delineating Γ*_s_* in that range.

To better determine the role of the in- and out-of-plane confinement on the lifetime, we investigate the spectral weight of the localized resonances in the *k*-space and compare this to the bulk band structure of copper. We calculate the wave function Ψ, which we assume to be separable, in the directions parallel and perpendicular to the (100) direction to obtain the corresponding *k*-space distribution. First, we consider the out-of-plane direction, where the confinement is set by the tip–sample distance and the applied voltage. We restrict our focus to the calculated wave function, Ψ(*z*), for the first principal FER and the resulting Fourier transform, Ψ(*k*_⊥_), shown in Figure 4a. The *k*_⊥_ values with a significant spectral weight span the entirety of the first Brillouin zone (BZ) (±1.75 Å).

Along the in-plane directions, we consider the wave functions Ψ(*x*) and Ψ(*y*) corresponding to the first ((1, 1)) particle-in-a-box mode for the 3 × 3 and 7 × 7 patches (Figure 4b, c). As expected, the *k*_∥_-space distribution widens as the patch size decreases. Furthermore, as shown in Figure 4c, this broadening also takes place when the quantum numbers (*n*_*x*_, *n*_*y*_) of the in-plane mode increase. This is due to changes in the apparent barrier height: Compare, for instance, the first and second particle-in-a-box modes. Since the latter lies at higher energy than the former, it experiences a shallower finite well. Such considerations allow us to visualize how the factors considered so far, such as the tip–sample distance, the lateral extent of the patch, and the apparent height of the in-plane barrier, impact the distribution of the state in *k*-space and consequently its overlap with the bulk states.

To better illustrate this, we consider the band structure of bulk copper along the high symmetry lines,^35^ specifically at the experimental energies of the particle-in-a-box modes (Figure 4d). The lifetime of the confined electrons depends directly on, and is limited by, the number of bulk states available for direct tunneling: The more bands we cross at the energy of the resonance, with *k*-values falling within Ψ(*k*), the shorter the lifetime to first-order. In this energy range, we cross several bulk bands along the high-symmetry lines (X → W, W → L, L → Γ, Γ → K); however, the efficiency of these decay paths is scaled by the spectral weight of Ψ(*k*) at the crossing points. In other words, the efficiency of the decay paths is scaled by the probability of having an electron with the right momentum for direct tunneling into that bulk state.

Accordingly, in Figure 4e, f, we consider the intensity of the *k*-space wave function along various cross sections of the first BZ (Figure 4d, inset). Interestingly, the highest spectral weight is along the Γ → X direction, across both the lateral (Figure 4e) and vertical (Figure 4f) cross sections, relative to the other high-symmetry lines; however, this direction does not present any band crossing along the high symmetry lines at the energy of the resonances. In fact, Ψ(*k*) carries little, if any, spectral weight along the other directions where it does cross the bulk bands. This is illustrated in Figure 4e, f, where we see that Ψ(*k*) has practically zero intensity along the energy isosurfaces (at 5 V and 6 V) of bulk Cu, calculated using density functional theory (DFT) (see Supporting Information section 5). This is quite counterintuitive: Although the lateral confinement of the states introduces direct tunneling paths to the bulk that are not present for the laterally freely propagating case, we can consider the contribution to be minimal in this case. Additionally, the added confinement acts to largely hinder the role of intraband inelastic scattering, as the available states for scattering are substantially reduced: The FERs no longer form bands, but rather, they are quantized and well-separated in energy, according to the physical dimensions of the patch. These two effects ultimately amount to a considerable enhancement of the lifetime of the confined states.

These considerations also shed light on the dependence of the sample decay rate Γ*_s_* with bias voltage, which as we previously found is critical in engendering NDR. With increasing voltage, the localized resonance is pushed to higher energies, causing a shift in the crossing points with the bulk bands. In turn, this shift translates into the decay channels being scaled by a slightly different spectral weight. To illustrate this effect, we can consider the crossing along the Γ → K direction: As the bias increases, the FER shifts up in energy, meaning that the crossing point for the lower band moves away from the Γ point, closer to the K point. Figure 4e, f shows that this shift is accompanied by a decrease in the spectral weight of Ψ(*k*), meaning the total overlap between the localized state and the bulk bands decreases. The emergence of the upper band around ∼4.5 V, however, further complicates the picture, illustrating that the overall rate of change of the decay rate is hard to estimate. However, by qualitatively considering the evolution of the *k*-space overlap, we can already grasp the complexity of the dependence of Γ_*s*_ on the bias voltage.

To get a quantitative estimate of the change in the sample decay rate, we calculate the weighted *k*-space wave function overlap for each DFT-calculated crossing point throughout the entire BZ, and relate that to a dimensionless sample-decay rate via Fermi’s golden rule (Figure 4g, see also Supporting Information section 5). For this, we consider the calculated *k*-space wave function of the 5 × 5 patch for the first (1, 1), second (2, 1), (1, 2), and fourth (3, 1), (1, 3) particle-in-a-box modes, the only states with nonzero intensity at the center of the patch (see Figures 1d and 2a). As shown in Figure 4g, we see that the calculated sample decay rate for all three states monotonically decreases, i.e., the overlap of Ψ(*k*) with the bulk bands decreases with increasing voltage so that dΓ*_s_*/d*V* is negative; the ratio of this rate of change to the intercept is in good agreement with our quantitative results from the double-barrier model (Figure 2). The sample decay rate associated with each state is strictly only applicable in the voltage range in which that state is measured, roughly delineated in Figure 4g by the shaded areas. All in all, we can confidently attribute the NDR to the effects of the bulk band structure. Additionally, we should also note that the NDR is consistently observed with different tips, and is not observed for laterally propagating FERs (see Figure S2),^36,37^ which do not have direct tunneling paths to the bulk available to them.

## Conclusions

By laterally confining field-emission resonances through atomic assembly of single chlorine vacancies, we present a platform for creating artificial atoms. We demonstrate control over the lifetime and occupation of these artificial atoms by adjusting the confining potential, implemented via modification of the tip–sample distance or the lateral dimensions of the patch. The ability to tune the occupation is a key parameter of control in the study of quantum many-body states that evolve as a function of the state filling. We show that the lifetime of field-emission resonances, unlike that of surface states, can be prolonged via lateral confinement, up to nearly four times the freely propagating case. This extension of the lifetime enhances the available energy resolution, and, in conjunction with control over the state filling, can carry implications for studying electron–electron interactions with artificial lattices. Further prolonging the lifetime to approach a state occupation of 1 for reasonable set point currents can be pursued via several avenues, such as finding an underlying bulk crystal that hosts FER bands closer to the Fermi energy or one that is semiconducting or even insulating. These considerations make confined vacuum resonances a promising platform for creating and studying artificial lattices.

## Methods

Sample preparation and experimentation were carried out in ultrahigh vacuum systems with a base pressure of 10^–10^ mbar (Unisoku USM1300s, SPECS Joule-Thompson-SPM). The Cu(100) crystal was cleaned via repeated cycles of argon sputter at 1 kV and annealing to 600 °C. The chlorinated copper surface was prepared by thermal evaporation (2–3 min) of anhydrous CuCl_2_ powder heated to 300 °C onto a warm Cu(100) crystal. The crystal was heated to 150 °C for ∼10 min before and after deposition.^23^ The coverage and sample quality were verified via LEED (where possible) and STM. Atom manipulation of chlorine vacancies was implemented using a procedure previously outlined.^23^ Differential conductance measurements were carried out using standard lock-in detection techniques.

## Supporting Information Available

Transport model for a double barrier potential, modeling the three-dimensional confining potential, corrections applied to stacked differential conductance spectra, estimating the sample decay rate (PDF)

## Notes

All data presented in this work are publicly available at https://doi.org/10.5281/zenodo.6473089.

## References

[ref1] Bloch I.; Dalibard J.; Nascimbène S. Quantum simulations with ultracold quantum gases. Nat. Phys. 2012, 8, 267–276. 10.1038/nphys2259.

[ref2] Bloch I. Ultracold quantum gases in optical lattices. Nat. Phys. 2005, 1, 23–30. 10.1038/nphys138.

[ref3] Gross C.; Bloch I. Quantum simulations with ultracold atoms in optical lattices. Science 2017, 357, 995–1001. 10.1126/science.aal3837. 28883070

[ref4] Blatt R.; Roos C. F. Quantum simulations with trapped ions. Nat. Phys. 2012, 8, 277–284. 10.1038/nphys2252.

[ref5] Khajetoorians A. A.; Wegner D.; Otte A. F.; Swart I. Creating designer quantum states of matter atom-by-atom. Nature Reviews Physics 2019, 1, 703–715. 10.1038/s42254-019-0108-5.

[ref6] Drost R.; Ojanen T.; Harju A.; Liljeroth P. Topological states in engineered atomic lattices. Nat. Phys. 2017, 13, 668–671. 10.1038/nphys4080.

[ref7] Slot M. R.; Gardenier T. S.; Jacobse P. H.; van Miert G. C. P.; Kempkes S. N.; Zevenhuizen S. J. M.; Smith C. M.; Vanmaekelbergh D.; Swart I. Experimental realization and characterization of an electronic Lieb lattice. Nat. Phys. 2017, 13, 672–676. 10.1038/nphys4105. 28706560 PMC5503127

[ref8] Gomes K. K.; Mar W.; Ko W.; Guinea F.; Manoharan H. C. Designer Dirac fermions and topological phases in molecular graphene. Nature 2012, 483, 306–310. 10.1038/nature10941. 22422264

[ref9] Gardenier T. S.; van den Broeke J. J.; Moes J. R.; Swart I.; Delerue C.; Slot M. R.; Smith C. M.; Vanmaekelbergh D. p orbital flat band and Dirac cone in the electronic honeycomb lattice. ACS Nano 2020, 14, 13638–13644. 10.1021/acsnano.0c05747. 32991147 PMC7596780

[ref10] Kempkes S. N.; Slot M. R.; van den Broeke J. J.; Capiod P.; Benalcazar W. A.; Vanmaekelbergh D.; Bercioux D.; Swart I.; Morais Smith C. Robust zero-energy modes in an electronic higher-order topological insulator. Nat. Mater. 2019, 18, 1292–1297. 10.1038/s41563-019-0483-4. 31548630

[ref11] Freeney S. E.; van den Broeke J. J.; Harsveld van der Veen A. J. J.; Swart I.; Morais Smith C. Edge-dependent topology in Kekulé Lattices. Phys. Rev. Lett. 2020, 124, 236404 10.1103/PhysRevLett.124.236404. 32603178

[ref12] Kempkes S. N.; Slot M. R.; Freeney S. E.; Zevenhuizen S. J. M.; Vanmaekelbergh D.; Swart I.; Smith C. M. Design and characterization of electrons in a fractal geometry. Nat. Phys. 2019, 15, 127–131. 10.1038/s41567-018-0328-0. 30886641 PMC6420065

[ref13] Huda M. N.; Kezilebieke S.; Ojanen T.; Drost R.; Liljeroth P. Tuneable topological domain wall states in engineered atomic chains. npj Quantum Materials 2020, 5, 17 10.1038/s41535-020-0219-3.

[ref14] Huda M. N.; Kezilebieke S.; Liljeroth P. Designer flat bands in quasi-one-dimensional atomic lattices. Phys. Rev. Research 2020, 2, 043426 10.1103/PhysRevResearch.2.043426.

[ref15] Girovsky J.; Lado J.; Kalff F.; Fahrenfort E.; Peters L.; Fernández-Rossier J.; Otte A. F. Emergence of quasiparticle Bloch states in artificial crystals crafted atom-by-atom. SciPost Physics 2017, 2, 020 10.21468/SciPostPhys.2.3.020.

[ref16] Collins L. C.; Witte T. G.; Silverman R.; Green D. B.; Gomes K. K. Imaging quasiperiodic electronic states in a synthetic Penrose tiling. Nat. Commun. 2017, 8, 15961 10.1038/ncomms15961. 28639623 PMC5489715

[ref17] Braun K. F.; Rieder K. H. Engineering electronic lifetimes in artificial atomic structures. Phys. Rev. Lett. 2002, 88, 096801 10.1103/PhysRevLett.88.096801. 11864039

[ref18] Jensen H.; Kröger J.; Berndt R.; Crampin S. Electron dynamics in vacancy islands: scanning tunneling spectroscopy on Ag(111). Phys. Rev. B 2005, 71, 155417 10.1103/PhysRevB.71.155417.

[ref19] Kliewer J.; Berndt R.; Crampin S. Scanning tunnelling spectroscopy of electron resonators. New J. Phys. 2001, 3, 22–22. 10.1088/1367-2630/3/1/322.

[ref20] Slot M. R.; Kempkes S. N.; Knol E. J.; van Weerdenburg W. M. J.; van den Broeke J. J.; Wegner D.; Vanmaekelbergh D.; Khajetoorians A. A.; Morais Smith C.; Swart I. p-Band engineering in artificial electronic lattices. Phys. Rev. X 2019, 9, 011009 10.1103/PhysRevX.9.011009.

[ref21] Freeney S.; Borman S.; Harteveld J.; Swart I. Coupling quantum corrals to form artificial molecules. SciPost Phys. 2020, 9, 85 10.21468/SciPostPhys.9.6.085.

[ref22] Jian Ping Sun; Haddad G.I.; Mazumder P.; Schulman J.N. Resonant tunneling diodes: models and properties. Proceedings of the IEEE 1998, 86, 641–660. 10.1109/5.663541.

[ref23] Kalff F. E.; Rebergen M. P.; Fahrenfort E.; Girovsky J.; Toskovic R.; Lado J. L.; Fernández-Rossier J.; Otte A. F. A kilobyte rewritable atomic memory. Nat. Nanotechnol. 2016, 11, 926–929. 10.1038/nnano.2016.131. 27428273

[ref24] Gartland P. O.; Berge S.; Slagsvold B. J. Photoelectric work function of a copper single crystal for the (100), (110), (111), and (112) faces. Phys. Rev. Lett. 1972, 28, 738–739. 10.1103/PhysRevLett.28.738.

[ref25] Westphal D.; Goldmann A. Chlorine adsorption on copper: II. Photoemission from Cu(001)c(2 × 2)-Cl and Cu(111)(√3 × √3)R30°-Cl. Surf. Sci. 1983, 131, 113–138. 10.1016/0039-6028(83)90122-X.

[ref26] Wahl P.; Schneider M. A.; Diekhöner L.; Vogelgesang R.; Kern K. Quantum coherence of image-potential states. Phys. Rev. Lett. 2003, 91, 106802 10.1103/PhysRevLett.91.106802. 14525497

[ref27] Ruffieux P.; Aït-Mansour K.; Bendounan A.; Fasel R.; Patthey L.; Gröning P.; Gröning O. Mapping the electronic surface potential of nanostructured surfaces. Phys. Rev. Lett. 2009, 102, 086807 10.1103/PhysRevLett.102.086807. 19257772

[ref28] Jung T.; Mo Y. W.; Himpsel F. J. Identification of metals in scanning tunneling microscopy via image states. Phys. Rev. Lett. 1995, 74, 1641–1644. 10.1103/PhysRevLett.74.1641. 10059080

[ref29] Pivetta M.; Patthey F.; Stengel M.; Baldereschi A.; Schneider W. D. Local work function Moiré pattern on ultrathin ionic films: NaCl on Ag(100). Phys. Rev. B 2005, 72, 115404 10.1103/PhysRevB.72.115404.

[ref30] Ploigt H. C.; Brun C.; Pivetta M.; Patthey F.; Schneider W.-D. Local work function changes determined by field emission resonances: NaCl/Ag(100). Phys. Rev. B 2007, 76, 195404 10.1103/PhysRevB.76.195404.

[ref31] Berthold W.; Höfer U.; Feulner P.; Chulkov E. V.; Silkin V. M.; Echenique P. M. Momentum-resolved lifetimes of image-potential states on Cu(100). Phys. Rev. Lett. 2002, 88, 056805 10.1103/PhysRevLett.88.056805. 11863766

[ref32] Echenique P.; Pitarke J.; Chulkov E.; Rubio A. Theory of inelastic lifetimes of low-energy electrons in metals. Chem. Phys. 2000, 251, 1–35. 10.1016/S0301-0104(99)00313-4.

[ref33] Crampin S. Lifetimes of Stark-shifted image states. Phys. Rev. Lett. 2005, 95, 046801 10.1103/PhysRevLett.95.046801. 16090830

[ref34] Echenique P.; Berndt R.; Chulkov E.; Fauster T.; Goldmann A.; Höfer U. Decay of electronic excitations at metal surfaces. Surf. Sci. Rep. 2004, 52, 219–317. 10.1016/j.surfrep.2004.02.002.

[ref35] Burdick G. A. Energy band structure of copper. Phys. Rev. 1963, 129, 138–150. 10.1103/PhysRev.129.138.

[ref36] Pascual J. I.; Corriol C.; Ceballos G.; Aldazabal I.; Rust H.-P.; Horn K.; Pitarke J. M.; Echenique P. M.; Arnau A. Role of the electric field in surface electron dynamics above the vacuum level. Phys. Rev. B 2007, 75, 165326 10.1103/PhysRevB.75.165326.

[ref37] Stepanow S.; Mugarza A.; Ceballos G.; Gambardella P.; Aldazabal I.; Borisov A. G.; Arnau A. Localization, splitting, and mixing of field emission resonances induced by alkali metal clusters on Cu(100). Phys. Rev. B 2011, 83, 115101 10.1103/PhysRevB.83.115101.

[ref38] Rejali R.; Farinacci L.; Otte S. Normalization procedure for obtaining the local density of states from high-bias scanning tunneling spectroscopy. arXiv (Mesoscale and Nanoscale Physics), April 25, 2022, 2204.09929, ver. 2, https://arxiv.org/pdf/2204.09929.

