# Supplementary material for: Confined Vacuum Resonances as Artificial Atoms with Tunable Lifetime
Source: ACS Nano. 2022 Jul 11;16(7):11251–8. doi: 10.1021/acsnano.2c04574 (PMC9331178; doi:10.1021/acsnano.2c04574)
Supplement: Supplementary file 1 — nn2c04574_si_001.pdf [file nn2c04574_si_001.pdf]

Supplementary information: Confined vacuum resonances as artificial atoms with tunable lifetime

Rasa Rejali, Laëtitia Farinacci, David Coffey, Rik Broekhoven, Jeremie Gobeil, Yaroslav M. Blanter, and Sander Otte

Department of Quantum Nanoscience, Kavli Institute of Nanoscience, Delft University of Technology, Lorentzweg 1, Delft, 2628 CJ, The Netherlands

1. MODELLING THE TRANSPORT THROUGH A DOUBLE-BARRIER POTENTIAL

Transport through the double potential barrier is dictated by the voltage and height-dependent tip and sample decay rates, Γ_s and Γ_t, respectively. The transmission coefficient for resonant tunneling through a single level localized between the barriers at an energy E_i is given by the Breit-Wigner formula [1]:

$$T_{\mathrm{BW}}(E,z,V) = \frac{\Gamma_t(E,z,V)\Gamma_s(E,z,V)}{((\Gamma_t(E,z,V)+\Gamma_s(E,z,V))/2)^2 + ((E-E_i(z,V))/\hbar)^2}. \tag{S1}$$

The energy of the ith level E_i is strictly a function of voltage: the state shifts higher in energy with increasing bias voltage. We can account for this change using the Bohr-Sommerfield quantization condition in the WKB approximation of a triangular potential well $\oint p(z')dz' = 2\pi\hbar(i+3/4)$, where the momentum is defined by $p(z') = \sqrt{(2m(E-V(z'))}$, and the potential is $V(z') = (\phi_t - \phi_s + eV)z'/z$, where z is the total width of the barrier (the tip-sample separation). See Fig. 2 (inset) of the main manuscript for a schematic of the potential considered. By integrating the absolute value of the momentum from the start of the well ($z'=0$) to the classical turning point z_{tp}, we find the analytical form for the discrete levels:

$$E_i(z,V) = \left(\frac{3\pi\hbar}{2}\frac{eV+\phi_t-\phi_s}{z}\left(i+\frac{3}{4}\right)\right)^{2/3}(2m)^{-1/3}. \tag{S2}$$

Furthermore, we can relate the tip decay rate to the WKB transmission coefficient through the tunnel barrier via a proportionality constant. To estimate this transmission, we consider that in the field emission regime, the applied voltage is necessarily greater than the sample work function; as such, we can approximate the tunnel barrier to be triangular, in the region where $eV > \phi_s$, with a transmission coefficient given by $\mathcal{T}(z,V,E) = \exp\left(\frac{-2}{\hbar}\int |p(z')|dz'\right)$:

$$-\frac{2}{\hbar}\int_{z_0}^{z}|p(z')|dz' = -\frac{2}{\hbar}\int_{z_0}^{z}\sqrt{2m_e\left(\phi_s + \frac{\phi_t-\phi_s+eV}{z}z' - E\right)}dz', \tag{S3}$$

where the classical turning point is denoted by z_0, the width of the barrier is given by the tip-sample distance z, and ϕ_s and ϕ_t are the tip and sample work functions, respectively. By substitution:

$$\alpha = \phi_s + \frac{\phi_t-\phi_s+eV}{z}z' - E \qquad d\alpha = \frac{\phi_t-\phi_s+eV}{z}dz',$$

$$\begin{aligned}
-\frac{2}{\hbar}\int_{z_0}^{z}p(z')dz' &= -\frac{2\sqrt{2m_e}}{\hbar}\int_{\phi_s+\frac{\phi_t-\phi_s+eV}{z}z_0-E}^{\phi_t+eV-E}\frac{z}{\phi_t-\phi_s+eV}\sqrt{\alpha}d\alpha \\
&= -\frac{4\sqrt{2m_e}}{3\hbar}\frac{z}{\phi_t-\phi_s+eV}\left((\phi_t+eV-E)^{3/2} - \left(\phi_s+\frac{\phi_t-\phi_s+eV}{z}z_0-E\right)^{3/2}\right) \\
&= -\frac{4\sqrt{2m_e}}{3\hbar}\frac{z}{\phi_t-\phi_s+eV}(\phi_t+eV-E)^{3/2}.
\end{aligned}$$

From this we can derive a transmission coefficient for the tunnel barrier [2]:

$$\mathcal{T}(z,V,E) = \exp\left(-\frac{4\sqrt{2m_e}}{3\hbar}\frac{z}{\phi_t-\phi_s+eV}(\phi_t+eV-E)^{3/2}\right). \tag{S4}$$

As mentioned, we can relate this to the tip decay rate via the proportionality constant λ:

$$\Gamma_t(z,V,E=E_i(z,V)) = \lambda\exp\left(-\frac{4\sqrt{2m_e}}{3\hbar}\frac{z}{\phi_t-\phi_s+eV}(\phi_t+eV-E_i(z,V))^{3/2}\right), \tag{S5}$$

where, for simplicity, we consider the tip decay rate only at the energy of the bound state E_i. By using Eq. S2 and focusing only on the first bound state ($i=0$), we can determine the energy $E_0 = \left(\frac{9\pi\hbar}{8}\frac{eV+\phi_t-\phi_s}{z\sqrt{2m}}\right)^{2/3}$. The explicit form of the tip decay rate in this case is:

$$\Gamma_t(z,V,E=E_0(z,V)) = \lambda\exp\left(-\frac{4\sqrt{2m_e}}{3\hbar}\frac{z}{\phi_t-\phi_s+eV}\left(\phi_t+eV-\left(\frac{9\pi\hbar}{8}\frac{eV+\phi_t-\phi_s}{z\sqrt{2m}}\right)^{2/3}\right)^{3/2}\right). \tag{S6}$$

To determine the current, we integrate the Breit-Wigner transmission, assuming the zero-temperature limit and positioning the chemical potentials of the sample and the tip at 0 and eV, respectively:

$$I = \frac{G_Q}{e}\int_0^{eV} T_{\mathrm{BW}}(E,z,V)dE \tag{S7}$$

$$= \frac{2G_Q\hbar}{e}\frac{\Gamma_t(z,V)\Gamma_s(z,V)}{(\Gamma_t(z,V)+\Gamma_s(z,V))}\left(\tan^{-1}\left(\frac{2(eV-E_i(z,V))}{\hbar(\Gamma_t(z,V)+\Gamma_s(z,V))}\right) + \tan^{-1}\left(\frac{2E_i(z,V)}{\hbar(\Gamma_t(z,V)+\Gamma_s(z,V))}\right)\right), \tag{S8}$$

where G_Q is the conductance quantum $e^2/(\pi\hbar)$ (accounting for the spin degeneracy). If the levels are resolvable, namely that $E_i \gg \Gamma_t(z,V)+\Gamma_s(z,V)$, then the expression for the current simplifies to:

$$I = \frac{2G_Q\hbar}{e}\frac{\Gamma_t(z,V)\Gamma_s(z,V)}{\Gamma_t(z,V)+\Gamma_s(z,V)}\left(\frac{\pi}{2}+\tan^{-1}\left(\frac{2(eV-E_i(z,V))}{\hbar(\Gamma_t(z,V)+\Gamma_s(z,V))}\right)\right), \tag{S9}$$

In general, the differential conductance depends on the partial derivatives of $\Gamma_t(z,V)$, $\Gamma_s(z,V)$, and $E_i(z,V)$ with respect to voltage; for ease of notation, we will refer to these partial derivatives as $\dot{\Gamma}_t$, $\dot{\Gamma}_s$, and $\dot{E}_i$, and drop the explicit reference to their dependence on z and V. The differential conductance can be evaluated accordingly:

$$\frac{\mathrm{d}I}{\mathrm{d}V} = \frac{2G_Q\hbar}{e}\left[\left(\frac{\dot{\Gamma}_t\Gamma_s+\Gamma_t\dot{\Gamma}_s}{\Gamma_t+\Gamma_s} - \frac{\Gamma_t\Gamma_s(\dot{\Gamma}_t+\dot{\Gamma}_s)}{(\Gamma_t+\Gamma_s)^2}\right)\left(\frac{\pi}{2}+\arctan\frac{eV-E_i}{\Gamma_t+\Gamma_s}\right) + \frac{2\hbar\Gamma_t\Gamma_s}{\Gamma_t+\Gamma_s}\frac{(E_i-eV)(\dot{\Gamma}_t+\dot{\Gamma}_s)-(\Gamma_t+\Gamma_s)\dot{E}_i+e(\Gamma_s+\Gamma_t)}{\hbar^2(\Gamma_t+\Gamma_s)^2+4(E_i-eV)^2}\right]. \tag{S10}$$

The second term in the sum in the above expression corresponds to a Lorentzian lineshape centered at E_i, with width and amplitude determined by Γ_t and Γ_s. We can use the analytical forms of the tip decay rate and the changing level of the resonance to explicitly evaluate the respective derivatives:

$$\dot{\Gamma}_t = \frac{4\sqrt{2m_e}}{3\hbar}\frac{z(\phi_t+eV-E_i)^{1/2}}{\phi_t-\phi_s+eV}\left(\frac{e(\phi_t+eV-E_i)}{\phi_t-\phi_s+eV}-\frac{3}{2}\left(e-\dot{E}_i\right)\right)\Gamma_t(z,V), \tag{S11}$$

and

$$\dot{E}_i = \frac{\pi\hbar e}{\sqrt{2m}z}\left(i+\frac{3}{4}\right)\left(\frac{3\pi\hbar}{2}\frac{eV+\phi_t-\phi_s}{z\sqrt{2m}}\left(i+\frac{3}{4}\right)\right)^{-1/3}. \tag{S12}$$

Finally, in this framework, the time-average occupation p of the confined level is given by the ratio of the tip and sample decay rates at the energy of the bound state:

$$p = \frac{\Gamma_t(E_i)}{\Gamma_s(E_i)+\Gamma_t(E_i)}. \tag{S13}$$

A. Generalization to more than one independent level

If there is more than one independent level within the energy range of interest, then the total current is defined by the sum of the current through each level. In other words, Eq. S9 assumes the form:

$$I_i = \frac{2G_Q\hbar}{e}\frac{\Gamma_t^i(z,V)\Gamma_s^i(z,V)}{\Gamma_t^i(z,V)+\Gamma_s^i(z,V)}\left(\frac{\pi}{2}+\tan^{-1}\left(\frac{2(eV-E_i(z,V))}{\hbar(\Gamma_t^i(z,V)+\Gamma_s^i(z,V))}\right)\right), \tag{S14}$$

where we have defined the current through the ith level according to its energy, E_i, and the tip and sample decay rates, Γ_t^i and Γ_s^i. The total current is therefore:

$$I = \sum_i I_i \tag{S15}$$

B. Simplification of the differential conductance for fitting a single resonance

As mentioned above, the expression of the differential conductance (considering only a single level, see Eq. S10) accounts for the voltage-dependence of Γ_t, Γ_s, and E_i, resulting in a rather unwieldy form. To simplify this further, we estimate the contributions of the terms containing $\dot{\Gamma}_t$, $\dot{\Gamma}_s$, and $\dot{E}_i$ by modelling the measured dI/dV (following the procedure outlined in section 1C) in two ways: either by considering the full expression (Eq. S10), or a simplified one that only accounts for the explicit derivative of the current with respect to V:

$$\frac{\mathrm{d}I}{\mathrm{d}V} \approx \frac{4\hbar^2 G_Q\Gamma_t\Gamma_s}{\hbar^2(\Gamma_t+\Gamma_s)^2+4(E_i-eV)^2}. \tag{S16}$$

To do so, we consider the first field-emission resonance on a bare Cu(100) surface (see figure S1), and model the measured differential conductance with the full and simplified expressions: the resulting fit parameters vary by $< 4\%$, and the qualitative features of the fit are in good agreement around the peak. Thus, we justify using this simplified expression (Eq. S16) in fitting the first resonance for estimations of the lifetime. We note here that the full expression (Eq. S10) must be used for fitting the entirety of the differential conductance spectrum (consisting of several independent peaks, over a broad voltage range), as the additional terms cannot be neglected beyond the limited scope of a single peak.

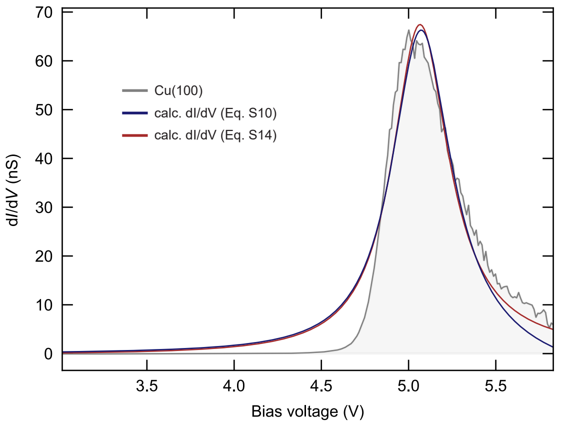

Fig. S1. Comparison of the simplified (Eq. S16, red line) and full (Eq. S10, blue line) expressions for the differential conductance, used to fit differential conductance spectroscopy obtained at 4 K atop bare Cu(100), at a conductance set-point of 32 nA, 5.8 V.

C. Modelling the measured dI/dV

By putting together Eqs. S2, S10, and S5, we can model the measured differential conductance using our analytical framework. We implement the dependence of the level on the applied voltage and tip-sample distance (Eq. S2) according to $E_i = \beta_i(eV/z)^{2/3}$, where β_i is a fit parameter; for

simplicity, we assume the work functions of the tip and sample are the same. This assumption is justified on two fronts: first, the work functions of the copper crystal and tungsten tip are similar (4.6 eV and 4.5 eV, respectively); second, our tip preparation methods, which involve dipping the tip into the surface (meaning it is likely partially covered in copper), bring the two work functions closer together. We apply the same assumption to equation S5; additionally, we take λ as a fit parameter in defining the tip decay rate. The tip-sample distance z is fixed according to the relative displacement of the z-piezo at different conductance set-points for each patch, with a universal offset parameter (to transform the relative displacement to a total tip-sample separation) that is obtained by fitting the spectrum taken at closest tip-sample distance. We have confirmed that large variations (± 1 nm) in this universal offset do not impact the lifetime values. As for the sample decay rate, we take two approaches: for fitting the first principle resonance only (in which we use the approximate differential conductance in Eq. S16), we assume that Γ_s is a constant, and we take this as a fitting parameter. Conversely, for fitting the main resonance and the subsequent confinement modes simultaneously (for which we use the full form of the differential conductance in Eq. S10), we assume that $\Gamma_s = aV + b$, where a and b are fit parameters.

D. Estimation of the lifetime of the first resonance on bare Cu(100)

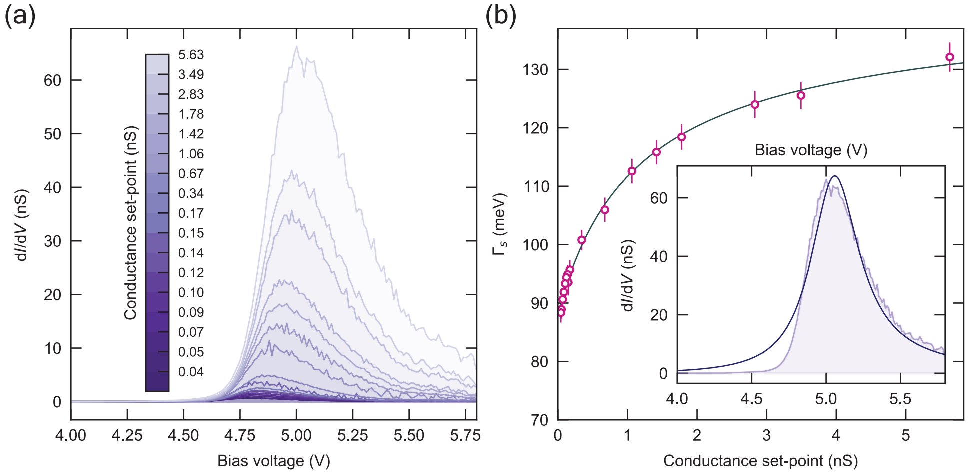

Fig. S2. Determining the lifetime of the first field emission resonance of Cu(100) at 4 K. (a) Constant-height differential conductance measurements acquired for bare Cu(100) for a range of conductance set-points. (b) Inset: modelled $\mathrm{d}I/\mathrm{d}V$ (dark blue) compared to constant-height measurement (purple, filled) at a conductance set-point of 32 nA, 5.8 V. The sample decay rate derived from the modelled $\mathrm{d}I/\mathrm{d}V$ as a function of conductance set-point at 4 K, yielding a lifetime of 8 ± 3 fs.

We benchmark our proposed model for determining the lifetime by applying it to first field-emission resonance on Cu(100). The lifetime of this state has previously been determined by studying the scattering of FER electrons at step edges on the surface, and relating that to a phase coherence length, which in turn can be readily converted into a lifetime [3]. Here, we fit the measured constant-height differential conductance spectrum using our rate-equations model (specifically using the simplified expression for the differential conductance, Eq. S16) to extract a sample decay rate, Γ_s, as function of conductance set-point, shown in figure S2.

An inverse natural logarithm fit to Γ_s versus conductance allows us to extract a value for Γ_s at zero conductance set-point, Γ_{s_0}, which is related to the lifetime via $\tau_{\mathrm{Cu}(100)} = \hbar/\Gamma_{s_0}$. This yields a lifetime of 8 ± 3 fs at 4 K, in fair agreement with previously measured lifetimes obtained at 6 K [3, 4]

2. MODELLING THE IN-PLANE CONFINEMENT

The engineered lateral confinement of the field emission resonances, which physically arises from the work function difference between the bare and chlorinated Cu(100) surfaces, can be simply modelled using a finite, slanted square potential well. The depth of the potential well is set by

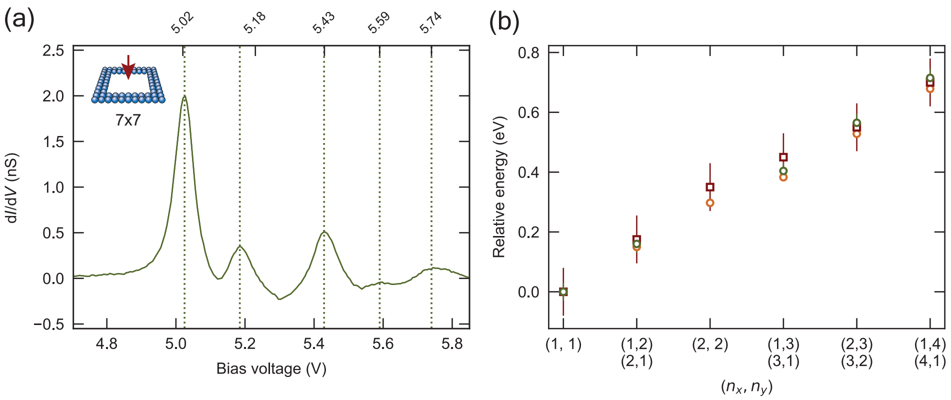

Fig. S3. Comparison of calculated and measured eigenenergies of the particle-in-a-box states of the laterally confined field-emission resonances. (a) Constant-height differential conductance spectrum (conductance set-point at 250 pA/6.2 V) acquired at the center of the 7×7 patch, as indicated in the inset. Dotted lines indicate the energy of the first main resonance (5.02V) and the following sub-resonances. (b) Resonance energies extracted from point spectroscopy performed at the center of the 7×7 patch (green circles); we compare this to the energies of the constant-current (100 pA) differential conductance maps (red squares) shown in figure 1e of the main text, as well as the energy calculated for each state using a finite potential well model (orange circles). In each case, we define the energy axis relative to the energy of the first field-emission resonance.

the work function difference $\Delta\phi = 1.1 \pm 0.1$ eV between the two surfaces [5]. It is also possible to estimate $\Delta\phi$ from the energy shift of the first field-emission resonance on each surface [6, 7]; this method also yields a difference of roughly 1 V (see figure 1c of the main manuscript). Since the work function change cannot occur with an infinitely sharp slope, we assume the potential well is slanted: we consider that the slope is set by $\Delta\phi$, as well as by the Fermi wavelength of the tunneling electrons, which is roughly 1.5 units cells at 5V.

We calculate the expected energy of the observed resonances by numerically solving the Schrödinger equation [8] for this potential; this gives us the energy spacing between the main and sub resonances. The total potential landscape, which also has an out-of-plane component, described by the trapezoidal potential barrier at the tip-sample junction, is separable, meaning the eigenenergies for each direction can be simply added. For ease of comparison between the energies calculated for the in-plane confinement and those measured, we subtract the out-of-plane component by defining the energy axis relative to the energy of the first field-emission resonance. As shown in figure S3, the comparison between the energies extracted from the measured constant-height $\mathrm{d}I/\mathrm{d}V$ to those obtained numerically is fair. Note that the third in-plane confinement mode is absent in the measured $\mathrm{d}I/\mathrm{d}V$ spectrum obtained at the center of the patch, as this point corresponds to the intersection of the two nodal planes for the $(n_x, n_y) = (2, 2)$ state. As such, we also compare the results of the calculations to the energies of the constant-current $\mathrm{d}I/\mathrm{d}V$ maps shown in figure 1e of the main text—again, we have good agreement.

3. MODELLING THE OUT-OF-PLANE CONFINEMENT

The field emission resonances are modelled with a one-dimensional potential [9, 10]. The sample potential ($z \leq 0$) is taken to be periodic in the bulk, with a periodicity set by $a/2$, the distance between two atomic layers in the out-of-plane direction, where a is the lattice constant. The potential beyond the surface atomic layer is modelled with a potential well ($0 \leq z \leq z_1$) and subsequently, an exponential decay of the potential towards the tip vacuum level. ($z_1 \leq z \leq z_{\mathrm{im}}$). To account for the tip (at z_{tip}) [9], we add the linear potential, $V_{\mathrm{lin}}(z)$, between the tip and sample ($z_1 \leq z \leq z_{\mathrm{tip}}$), caused by the applied voltage, and account for the contact potential by including the tip (ϕ_t) and sample work functions (ϕ_s). The long-range image potential, $V_{\mathrm{im}}(z)$, which accommodates multiple images in both tip and sample, is also included, giving rise to the

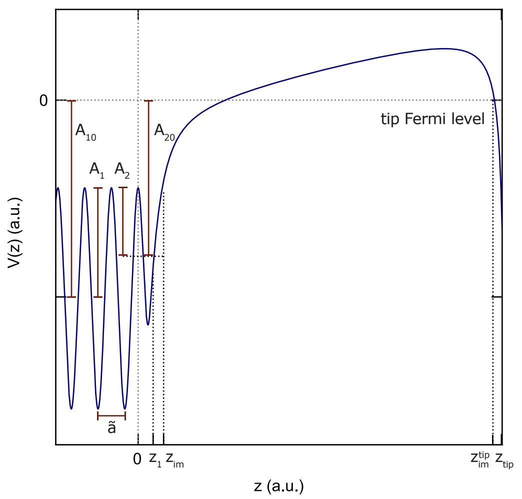

Fig. S4. Schematic of the total potential in the out-of-plane direction, with the relevant model parameters defined.

following total potential (figure S4) across the tip-sample junction:

$$V(z) = \begin{cases} A_{10} + A_1 \cos\left(\frac{2\pi}{\tilde{a}}\right) & z \leq 0 \\ A_{20} + A_2 \cos(\beta z) & 0 \leq z \leq z_1 \\ V_{\text{lin}}(z) + A_3 \exp(-\alpha(z - z_1)) & z_1 \leq z \leq z_{\text{im}} \\ V_{\text{lin}}(z) - V_{\text{im}}(z) & z_{\text{im}} \leq z \leq z_{\text{tip}}, \end{cases} \tag{S17}$$

where

$$V_{\text{lin}}(z) = E_{f,s} + s(eV + \phi_t) + (1 - s)\phi_s, \tag{S18}$$

given $E_{f,s}$ is the sample Fermi energy, and $s = (z - z_1)/(z_{\text{tip}} - z_1)$. We chose to define the potential relative to the tip Fermi level, meaning $E_{f,s} = -eV_{\text{bias}}$. Additionally, we can define the image potential as:

$$V_{\text{im}}(z) = (1 - \exp(-\lambda(z - z_{\text{im}})) \frac{e^2 (2\Psi(1) - \Psi(\eta) - \Psi(1-\eta))}{16\pi\epsilon_0 \left(z_{\text{im}}^{\text{tip}} - z_{\text{im}}\right)}, \tag{S19}$$

where $\eta = (z - z_{\text{im}})/(z_{\text{im}}^{\text{tip}} - z_{\text{im}})$, e the electron charge, ϵ_0 the vacuum permittivity, and Ψ is the digamma function.

For a terminated metal surface, the parameters A_1 and A_{10} determine the width and position of the surface-projected band gap, respectively, whereas the parameters A_2 and β reproduce the experimental values of the binding energies of the image stakes in the absence of the tip. We set $A_{10} = -eV_{\text{bias}} - A_1$, as we chose the tip vacuum level as our reference, and assign A_1, A_{20}, A_2, β, and $z_1 = 5\pi/(4\beta)$ to their corresponding values previously determined for a terminated Cu(100) surface, in the absence of the tip [10]. By forcing the potential and its derivative to be continuous everywhere (except at the tip position, z_{tip}), we can analytically determine the values for the remaining parameters (A_3, α, λ, z_{im}) in terms of already known parameters.

$$\begin{aligned} A_{20} &= A_{10} + A_1 - A_2 \\ A_3 &= A_{20} + A_2 \cos(\beta z_1) - V_{\text{lin}}(z_1) \\ \alpha &= \frac{eV_{\text{bias}} + \phi_t - \phi_s}{A_3(z_{\text{tip}} - z_1)} + \frac{\beta A_2}{A_3} \sin(\beta z_1) \\ \lambda &= 2\alpha \\ z_{\text{im}} &= \frac{-1}{\alpha} \ln\left(\frac{-\lambda e^2}{16\pi\epsilon_0 A_3}\right) + z_1 \end{aligned}$$

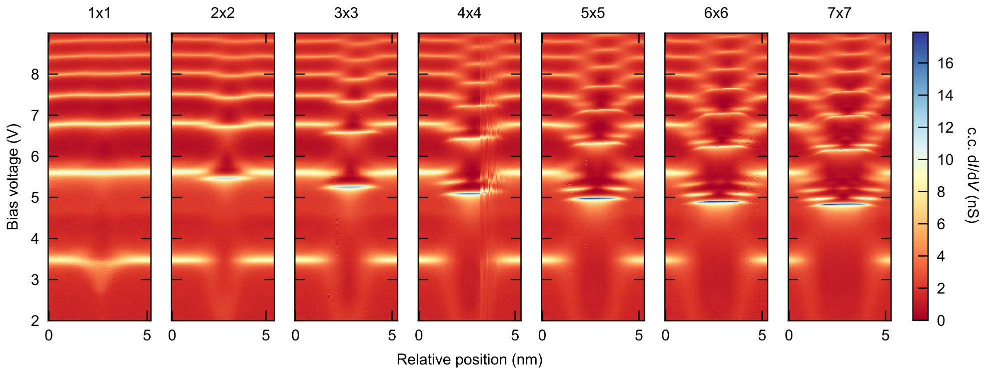

Fig. S5. Raw, uncorrected stacked constant-current differential conductance spectra obtained at a set-point of 100 pA, taken along a line crossing the center of each patch (respective size indicated at the top).

We note that the tip image plane is not well defined in the above potential (and hard to estimate from experiment); we assign $z_{\text{im}}^{\text{tip}} \approx z_{\text{tip}} - 0.3$ Å. Solving the time-independent Schrödinger equation [8] using this potential (equation S17) results in the calculated wave-functions shown in the figures 1 and 4 of the main manuscript.

4. CORRECTION APPLIED TO STACKED DIFFERENTIAL CONDUCTANCE SPECTRA

The raw constant-height spectroscopy acquired over each patch (figure S5) shows a general trend: a shift of the spectroscopic features to the right as the bias voltage is increased. We attribute this shift to the asymmetric geometry of the tip, which consequently gives rise to an asymmetric tip electric field. To correct for this, we assume that for a given bias voltage, the acquired spectrum should be symmetric and centered. We achieve this by applying a second-order polynomial fit to each constant-voltage line for each patch, and use the global maximum along each slice to set the center point. A Gaussian filter is applied to the obtained extremums, and a second-order polynomial fit is once again applied to this for the final correction. This correction is visible as an uniformly dark red area on the top right of the stacked differential conductance spectra in the main text.

5. MODELLING THE SAMPLE DECAY RATE DEPENDENCE ON BIAS

A. Estimating the sample decay rate

To first order, the sample decay rate for each field emission resonance follows Fermi's golden rule:

$$\Gamma_s = \frac{2\pi}{\hbar} | \langle f | H' | i \rangle |^2 \rho_{E_f}. \tag{S20}$$

The initial state $|i\rangle = \sum_{\mathbf{k}} \psi(\mathbf{k}) |\mathbf{k}\rangle \otimes |0\rangle$ denotes a filled FER with k-space distribution $\psi(\mathbf{k})$, and Cu in the ground-state; and the final state $|f\rangle = |0\rangle \otimes |\mathbf{k}'\rangle$ indicating an unfilled FER and the Cu in first excited state, at the energy of the FER. Note that we have introduced the notation $|\mathbf{k}\rangle = \hat{c}_{\mathbf{k}}^{\dagger} |0\rangle$, with $\hat{c}_{\mathbf{k}}^{\dagger}$ the fermionic creation operator for quasi momentum $\mathbf{k} = (k_x, k_y, k_z)$.

Since the decay is dominated by elastic scattering, the Hamiltonian connecting the initial and final states can be written as $H' = \sum_{\mathbf{k}} J \hat{c}_{\mathbf{k}'}^{\dagger} \hat{c}_{\mathbf{k}}$. Here, J is the coupling between the FER and sample; we assume J does not vary with $\mathbf{k}$. Finally, the density of states of the bulk sample is denoted by $\rho_{E_f} = \frac{1}{\mathcal{V}} \sum_m \delta(E_f - \epsilon_{m\mathbf{k}})$, with $\mathcal{V}$ the volume, m the Cu band index and $\epsilon_{m\mathbf{k}}$ the corresponding

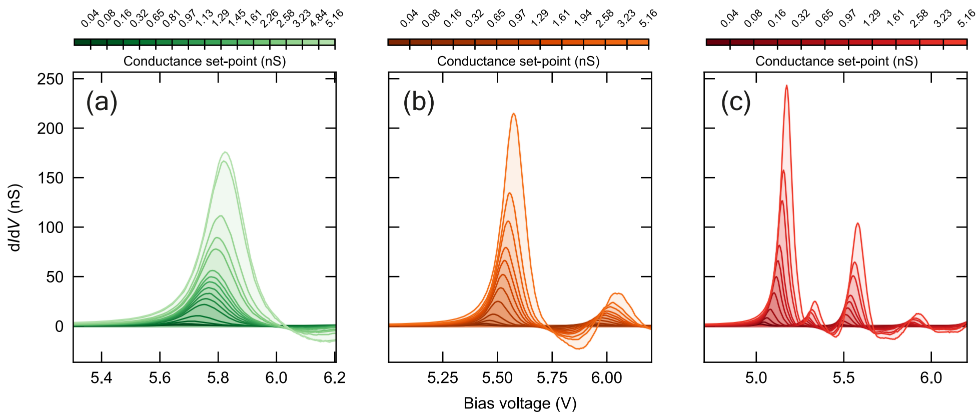

Fig. S6. Constant-height differential conductance spectra obtained at the center of the (a) 2×2, (b) 3×3, and (c) 7×7 patches for a range of conductance set-points ((250 pA $\rightarrow$ 32 nA), 6.2 V), indicated in the discretized color bar).

dispersion. When we substitute this in the above expression for the sample decay rate, we find:

$$\Gamma_s(V) = \frac{2\pi}{\hbar}\sum_{\mathbf{k}'} | \langle \mathbf{k}' | \langle 0 | J \sum_{\mathbf{k}\mathbf{k}'} c^\dagger_{\mathbf{k}'} c_{\mathbf{k}} |0\rangle \sum_{\mathbf{k}} \psi(\mathbf{k}) |\mathbf{k}\rangle |^2 \frac{1}{\mathcal{V}} \sum_m \delta(E_i(V) - \epsilon_{m\mathbf{k}}) \tag{S21}$$

$$= \frac{2\pi}{\hbar} J^2 \sum_{\mathbf{k}} |\psi(\mathbf{k})|^2 \frac{1}{\mathcal{V}} \sum_m \delta(E_i(V) - \epsilon_{m\mathbf{k}}) \tag{S22}$$

$$\propto \int_{BZ} d\mathbf{k} |\psi(\mathbf{k})|^2 \sum_m \delta(E_i(V) - \epsilon_{m\mathbf{k}}), \tag{S23}$$

where the proportionality comes from the constant $\frac{J^2}{2\pi\hbar}$. This expression can be simply understood as the overlap in k-space between the Cu band structure and the bound state, at the resonance energy of the latter. This energy (Eq. 1) depends on bias according to:

$$E_i(V) = \beta_i (eV)^{2/3}. \tag{S24}$$

Here, just like in section 1, we approximate the tip and sample work functions to be the same. We can determine β_i by assuming that the center of the resonance (measured via differential conductance spectroscopy) is equal to E_i at that bias V (see figure 2 of the main manuscript). Additionally, we assume the FER k-space distribution $\psi(\mathbf{k})$ remains approximately constant while the applied bias or tip-sample distance is changing. We can then evaluate the integral numerically using the Blöchl tetrahedron method for Brillouin zone (BZ) integration [11], as implemented in dfttools [12], with the Cu dispersion coming from DFT, and the FER wave function as calculated in sections 2 and 3. We note that prior to integration, we fold the part of the wave functions with finite weight outside the first BZ back onto the first BZ.

B. Density Functional Theory (DFT) calculations

To calculate the bulk band structure of Cu, we use plane-wave density-functional theory with a standard ultrasoft scalar relativistic pseudopotential and PBE exchange correlation functional, as implemented in the Quantum ESPRESSO package [13]. Plane wave energy cutoffs were set to 120/1080 Ry (wave function/density). We initialised the atoms on a FCC lattice with lattice constant 3.61 Å. Self-consistent calculation was done on a 4x4x4 k-point grid and followed by non self-consistent Gamma-point Brillouin zone sampling on a 32x32x32 k-point grid. The visualization of constant-energy surface cuts was done with FermiSurfer [14].

REFERENCES

1. Y. V. Nazarov and Y. M. Blanter, *Quantum Transport: Introduction to Nanoscience* (Cambridge University Press, Cambridge, 2009).

2. R. H. Fowler and L. Nordheim, "Electron emission in intense electric fields," Proc. Royal Soc. A **119**, 173–181 (1928).
3. P. Wahl, M. A. Schneider, L. Diekhöner, R. Vogelgesang, and K. Kern, "Quantum coherence of image-potential states," Phys. Rev. Lett. **91**, 106802 (2003).
4. S. Crampin, J. Kröger, H. Jensen, and R. Berndt, "Phase coherence length and quantum interference patterns at step edges," Phys. Rev. Lett. **95**, 029701 (2005).
5. D. Westphal and A. Goldmann, "Chlorine adsorption on copper: II. photoemission from Cu(001)c(2 $\times$ 2)-Cl and Cu(111)($\sqrt{3} \times \sqrt{3}$)R30°-Cl," Surf. Sci. **131**, 113–138 (1983).
6. H. C. Ploigt, C. Brun, M. Pivetta, F. Patthey, and W.-D. Schneider, "Local work function changes determined by field emission resonances: NaClAg(100)," Phys. Rev. B **76**, 195404 (2007).
7. M. Pivetta, F. Patthey, M. Stengel, A. Baldereschi, and W. D. Schneider, "Local work function Moiré pattern on ultrathin ionic films: NaCl on Ag(100)," Phys. Rev. B **72**, 115404 (2005).
8. C. W. Groth, M. Wimmer, A. R. Akhmerov, and X. Waintal, "Kwant: a software package for quantum transport," New J. Phys. **16**, 063065 (2014).
9. L. Limot, T. Maroutian, P. Johansson, and R. Berndt, "Surface-state stark shift in a scanning tunneling microscope," Phys. Rev. Lett. **91**, 196801 (2003).
10. E. Chulkov, V. Silkin, and P. Echenique, "Image potential states on metal surfaces: binding energies and wave functions," Surf. Sci. **437**, 330–352 (1999).
11. P. E. Blöchl, O. Jepsen, and O. K. Andersen, "Improved tetrahedron method for Brillouin-zone integrations," Phys. Rev. B **49**, 16223–16233 (1994).
12. A. Pulkin, "dfttools – parse, process and plot data from solid state and quantum chemistry applications," Zenodo, DOI: 10.5281/zenodo.6397700 (2022).
13. P. Giannozzi, O. Andreussi, T. Brumme, O. Bunau, M. B. Nardelli, M. Calandra, R. Car, C. Cavazzoni, D. Ceresoli, M. Cococcioni, N. Colonna, I. Carnimeo, A. D. Corso, S. de Gironcoli, P. Delugas, R. A. D. Jr, A. Ferretti, A. Floris, G. Fratesi, G. Fugallo, R. Gebauer, U. Gerstmann, F. Giustino, T. Gorni, J. Jia, M. Kawamura, H.-Y. Ko, A. Kokalj, E. Küçükbenli, M. Lazzeri, M. Marsili, N. Marzari, F. Mauri, N. L. Nguyen, H.-V. Nguyen, A. O. de-la Roza, L. Paulatto, S. Poncé, D. Rocca, R. Sabatini, B. Santra, M. Schlipf, A. P. Seitsonen, A. Smogunov, I. Timrov, T. Thonhauser, P. Umari, N. Vast, X. Wu, and S. Baroni, "Advanced capabilities for materials modelling with quantum espresso," J. Physics: Condens. Matter **29**, 465901 (2017).
14. M. Kawamura, "Fermisurfer: Fermi-surface viewer providing multiple representation schemes," Comput. Phys. Commun. **239**, 197–203 (2019).
